# Phylogenomics-Based Reconstruction and Molecular Evolutionary Histories of Brassica Photoreceptor Gene Families

**DOI:** 10.3390/ijms23158695

**Published:** 2022-08-04

**Authors:** Muthusamy Muthusamy, Jin-A Kim, Soo-In Lee

**Affiliations:** Department of Agricultural Biotechnology, National Institute of Agricultural Sciences (NAS), RDA, Jeonju 54874, Korea

**Keywords:** photoreceptor, Brassica, phytochrome, cryptochrome, phototropin, UVR8, F-box containing flavin binding proteins, gene evolution

## Abstract

Photosensory proteins known as photoreceptors (PHRs) are crucial for delineating light environments in synchronization with other environmental cues and regulating their physiological variables in plants. However, this has not been well studied in the Brassica genus, which includes several important agricultural and horticultural crops. Herein, we identified five major PHR gene families—phytochrome (PHY), cryptochrome (CRY), phototropin (PHOT), F-box containing flavin binding proteins (ZTL/FKF1/LKP2), and UV RESISTANCE LOCUS 8 (UVR8)—genomic scales and classified them into subfamilies based on their phylogenetic clustering with Arabidopsis homologues. The molecular evolution characteristics of Brassica PHR members indicated indirect expansion and lost one to six gene copies at subfamily levels. The segmental duplication was possibly the driving force of the evolution and amplification of Brassica PHRs. Gene replication retention and gene loss events of CRY, PHY, and PHOT members found in diploid progenitors were highly conserved in their tetraploid hybrids. However, hybridization events were attributed to quantitative changes in UVR8 and ZTL/FKF1/LKP2 members. All PHR members underwent purifying selection. In addition, the transcript expression profiles of PHR genes in different tissue and in response to exogenous ABA, and abiotic stress conditions suggested their multiple biological significance. This study is helpful in understanding the molecular evolution characteristics of Brassica PHRs and lays the foundation for their functional characterization.

## 1. Introduction

Plants have a series of unique photoreceptors (PHRs) with different wavelength absorption spectra and biochemical properties to delineate light environments precisely and regulate their physiological variables [1]. In the model plant *Arabidopsis thaliana*, five classes of PHRs, namely, phytochrome (PHY), cryptochrome (CRY), phototropin (PHOT), F-box containing flavin binding proteins (ZEITLUPE (ZTL)/LOV KELCH PROTEIN1 (LKP1), FLAVIN-BINDING KELCH REPEAT F-BOX 1 (FKF1), and LOV KELCH PROTEIN2 (LKP2)) and UV RESISTANCE LOCUS 8 (UVR8), control photoreception and corresponding light signaling networks. Plants optimize the light absorption through these PHRs, in synchronization with other environmental cues, as it is essential to balance photochemistry and photosynthesis reaction rates for the adaptive responses [2]. Of these, PHYs are useful in perceiving red/far-red lights (600–750 nm), while CRY, PHOT, and ZTL/FKF1/LKP2 are for detecting blue/UV-A light (320–500 nm) [3]. Similarly, UVR8 is a cytosol and nuclear-localized photoreceptor responsible for UV-B absorption and protecting plants from harmful effects of UV-B light (280–320 nm) [4]. The UVR8-mediated protective effect could partially come from induced flavonoid biosynthesis [5]. Besides enabling a photosynthetic lifestyle, these light-activated PHRs help plants guide various critical growth and developmental processes, including seed germination, flowering time, and the circadian clock [6]. Indeed, PHR gene expression patterns are significant in the daily regulation of plant physiology in higher plants [7]. PHRs absorb light signals through photoreceptive domains binding chromophores and convert them into a variety of biochemical signals, including activation of light response downstream gene expressions, as a part of a unique physiological response to the perceived light spectrum [8]. Plants use those perceived light signals of different wavelength to release the halted mRNA stored in RNA granules called processing bodies to quickly adapt to demanding specific environmental conditions [9]. Light-activated PHY, CRY, and UVR8 inhibit COP1 activity by binding the WD40 domain to enable photomorphogenic phenotypes in higher plants. COP1 is a crucial repressor of photomorphogenesis [10]. The genetic manipulation of PHRs can result in modification of agronomically beneficial traits, hence it is important to understand the molecular evolution and characterization of classical or novel members in this family [1,3,8].

PHYs are responsible for germination, shade avoidance response, and, together with cryptochromes, for de-etiolation, circadian entrainment, and flowering [11]. Besides, PHYs are crucial for CO_2_ uptake and biomass production in developing plants [12]. PHOTs are plasma membrane-associated photoreceptors and crucial for chloroplast positioning, phototropism [11], photosynthetic pigments composition, stomata conductance, and water-use efficiency in Arabidopsis [2]. The ZTL/FKF1/LKP2 members commonly encode for blue light-absorbing specialized period-ARNT-single-minded (PAS) domain/LOV (light, oxygen, or voltage) domain-containing proteins that regulate the circadian clock and flowering time [13]. The ZTL/FKF1/LKP2 possess highly conserved LOV (light, oxygen, or voltage), F-box, and Kelch-repeat domains direct circadian timing by proteasome-dependent degradation of clock proteins in plants [13,14,15]. CRYs are evolutionarily conserved, flavoprotein blue/ultraviolet light-sensing photoreceptors known to regulate seed germination, flowering time, and entrainment of the circadian clock [6], accumulation of anthocyanin, chlorophylls, and antioxidants [16], and blue-light dependent ROS production [17]. It is worth noting that the light environment is typically synchronized with other environmental signals, possibly indicating the chances of PHRs modulating the responses to a wide range of environmental cues suggesting a role as a “multi-sensor” [1]. In fact, AtPHYB enhances tolerance to drought stress by increasing the ABA sensitivity [18]. Similarly, CRYs are key regulators of abiotic stress responses, such as drought, salinity, heat, and high radiation (as reviewed in [19]). Due to the multiple significance of the PHR gene family in plant growth and crop improvement, identification and characterization of PHRs is necessary. However, it has not been well studied in the Brassica genus, which includes several important agricultural and horticultural crops. Apart from that, plants, being sessile organisms, can either add or reduce gene copies through evolutionary processes called gene duplication and fractionations to meet their environmental adaptation requirements. Hence understanding the molecular evolutionary histories of a gene family is necessary and will be a useful resource for identification of candidate genes and development of climate-resilient crops.

The Brassica genome has undergone whole-genome triplication (WGT)/triploidization since its divergence from the Arabidopsis lineage and then extensive diploidization to stabilize the genomes [20,21]. Among gene duplications, segmental and tandem duplication types are the primary driving forces in gene family expansion [22]. The WGT event in Brassica would have added three to six copies of orthologous genomic regions of *A. thaliana*. However, the substantial gene losses with significant disparity among triplicated genome segments result in variation in the number of members in the gene families. Meanwhile, the recent synteny analysis between diploid *B. rapa* and three Arabidopsis species showed that 5851 genes of *B. rapa* had no syntenic orthologues, suggesting the occurrence of WGT (polyploidy) and the subsequent genome stabilization process involving diploidization reshaped the genome structures of Brassica species [23]. The subsequent events of WGT, such as gene fractionation, duplication (segmental and tandem), and transpositions, are attributable to the contraction or expansion of a gene family with new genes and novel functionalities, which contributed to the remarkable morphological plasticity of Brassica species.

Therefore, to get insight into the evolution and expansion of five main PHR gene families—PHYs, CRYs, PHOTs, UVR8, and ZTL/FKF1/LKP2—were comprehensively and systematically analyzed in three diploids and two tetraploids of Brassica species. BLASTP and domain architecture of Arabidopsis homologues were considered for Brassica PHR gene family identification. The phylogenetic clustering of Brassica PHR gene members was used for family and subfamily classifications. Further, the gene structure, gene synteny between PHR members in a single genome, gene duplicates including segmental and tandem, chromosomal distribution pattern, and gene losses after WGT events, were analyzed to understand the evolutionary past and/or expansion histories of five major PHR gene families in Brassica. In addition, the transcript expression profiles of PHR genes across different tissue types (root, leaf, bud, silique, and callus) and in response to exogenous ABA application, abiotic (light, drought, salt, and cold), and biotic stress conditions (*Pectobacterium carotovorum* infection) suggested PHR genes play important roles in plant growth and development and stress responses. The presence of ABA-, stress-associated putative promoter motifs along with light response cis-elements further validate their multisensory functionality. The present study unearthed several new members of PHR genes, and the molecular evolutionary characteristics of PHR will be useful in further studies focusing on functional characterization of photoreceptor genes in Brassica species.

## 2. Results

### 2.1. Identification and Classification of Brassica PHR Gene Families

**Figure 1:**
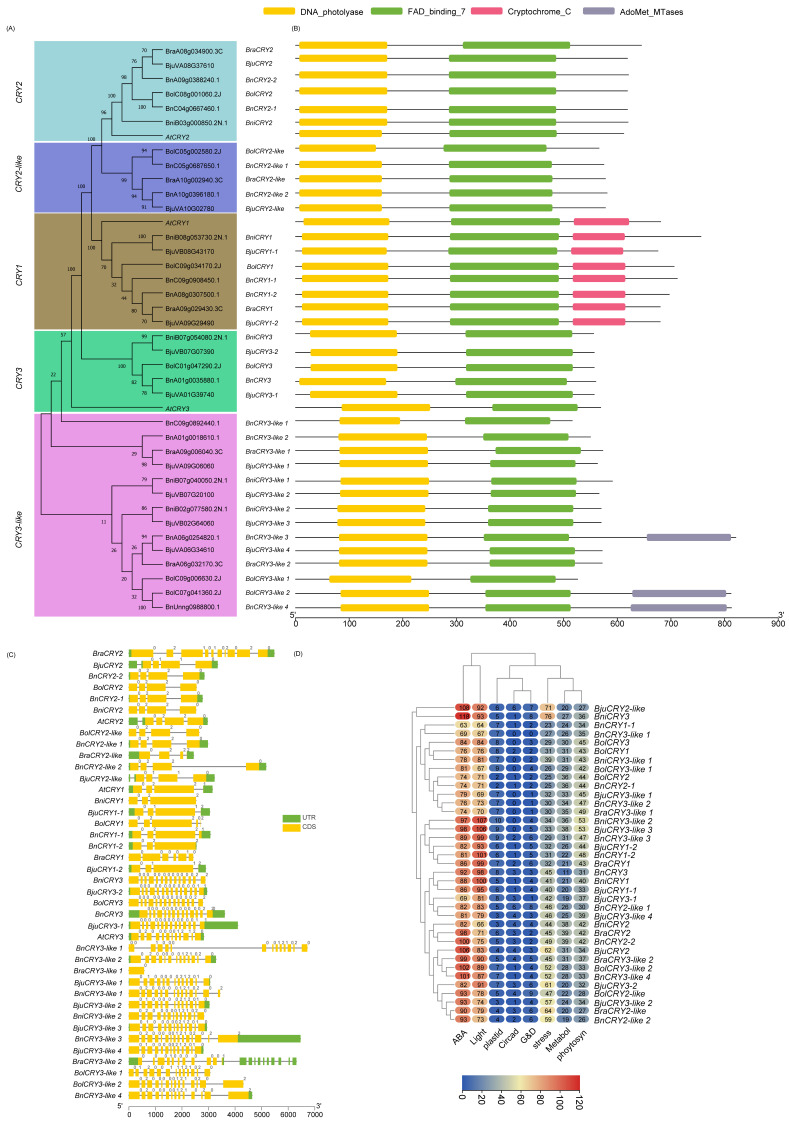
Phylogenetic analysis, classification, and structural description of cryptochrome (CRY) gene family in Brassica species. (**A**) The unrooted phylogenetic tree was established with Arabidopsis and five Brassica species (*B. rapa*, *B. oleracea*, *B. nigra*, *B. napus*, and *B. juncea*) CRY protein sequences using the neighbor-joining tree method with 1500 bootstrap replications in MEGA11. (**B**) Represents the domain architecture of phylogenetically classified CRY genes of Brassica species, while (**C**) represents the gene structures, including intron/exon pattern and intron phases (Class 0, 1, and 2) and UTRs. (**D**) Heatmap represents the key cis-regulatory elements (ABA- and light-responsive, elements associated with plastids, circadian rhythm, growth and development (G&D), stress, metabolite biosynthesis, and photosynthesis) and their abundance in putative promoter motifs of Brassica CRY genes reported in this study.

**Figure 2:**
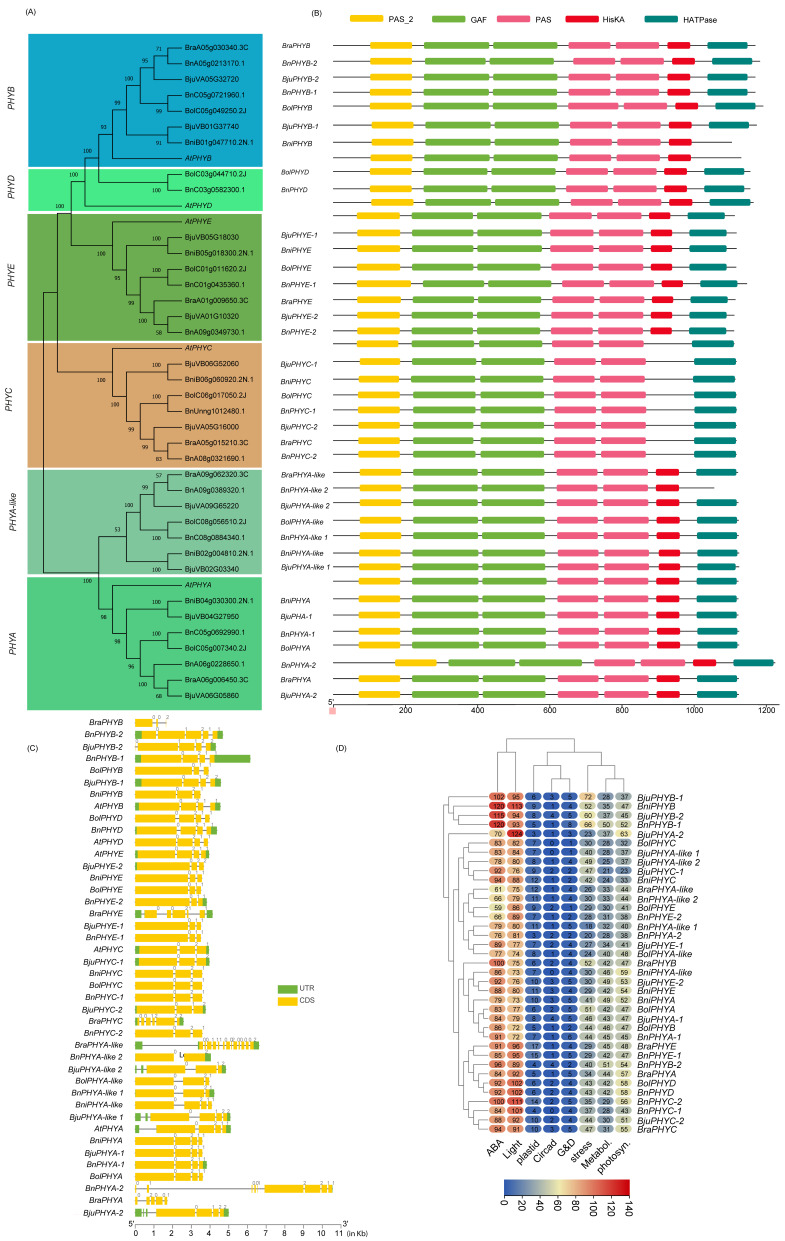
Phylogenetic analysis, classification, and structural description of phytochrome (PHY) gene family in Brassica species. (**A**) The unrooted phylogenetic tree was established with Arabidopsis and five Brassica species (*B. rapa*, *B. oleracea*, *B. nigra*, *B. napus*, and *B. juncea*) PHY protein sequences using the neighbor-joining tree method with 1500 bootstrap replications in MEGA11. (**B**) Represents the domain architecture of phylogenetically classified PHY genes of Brassica species, while (**C**) represents the gene structures including Intron/exon pattern and intron phases (Class 0, 1, and 2) and UTRs. (**D**) Heatmap represents the key cis-regulatory elements (ABA- and light-responsive, elements associated with plastids, circadian rhythm, growth and development (G&D), stress, metabolite biosynthesis, and photosynthesis) and their abundance in putative promoter motifs of Brassica PHY genes reported in this study.

**Figure 3:**
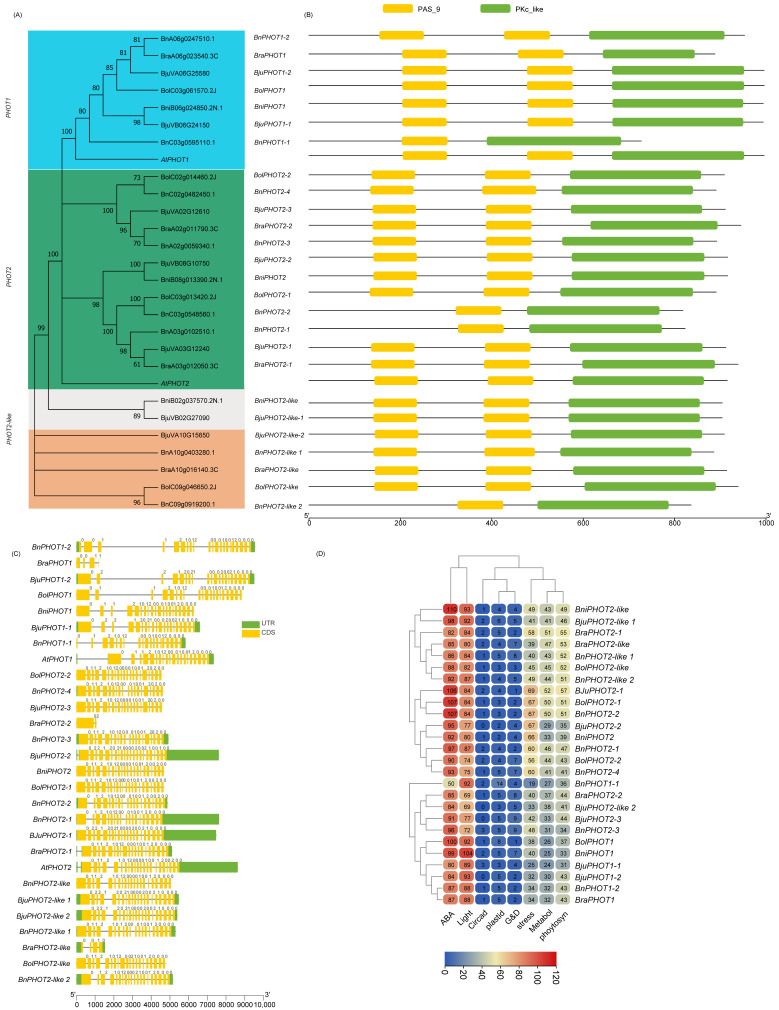
Phylogenetic analysis, classification, and structural description of phototropin (PHOT) gene family in Brassica species. (**A**) The unrooted phylogenetic tree was established with Arabidopsis and five Brassica species (*B. rapa*, *B. oleracea, B. nigra*, *B. napus*, and *B. juncea*) PHOT protein sequences using the neighbor-joining tree method with 1500 bootstrap replications in MEGA11. (**B**) Represents the domain architecture of phylogenetically classified PHOT genes of Brassica species, while (**C**) represents the gene structures including Intron/exon pattern and intron phases (Class 0, 1, and 2) and UTRs. (**D**) Heatmap represents the key cis-regulatory elements (ABA- and light-responsive, elements associated with plastids, circadian rhythm, growth and development (G&D), stress, metabolite biosynthesis, and photosynthesis) and their abundance in putative promoter motifs of Brassica PHOT genes reported in this study.

**Figure 4:**
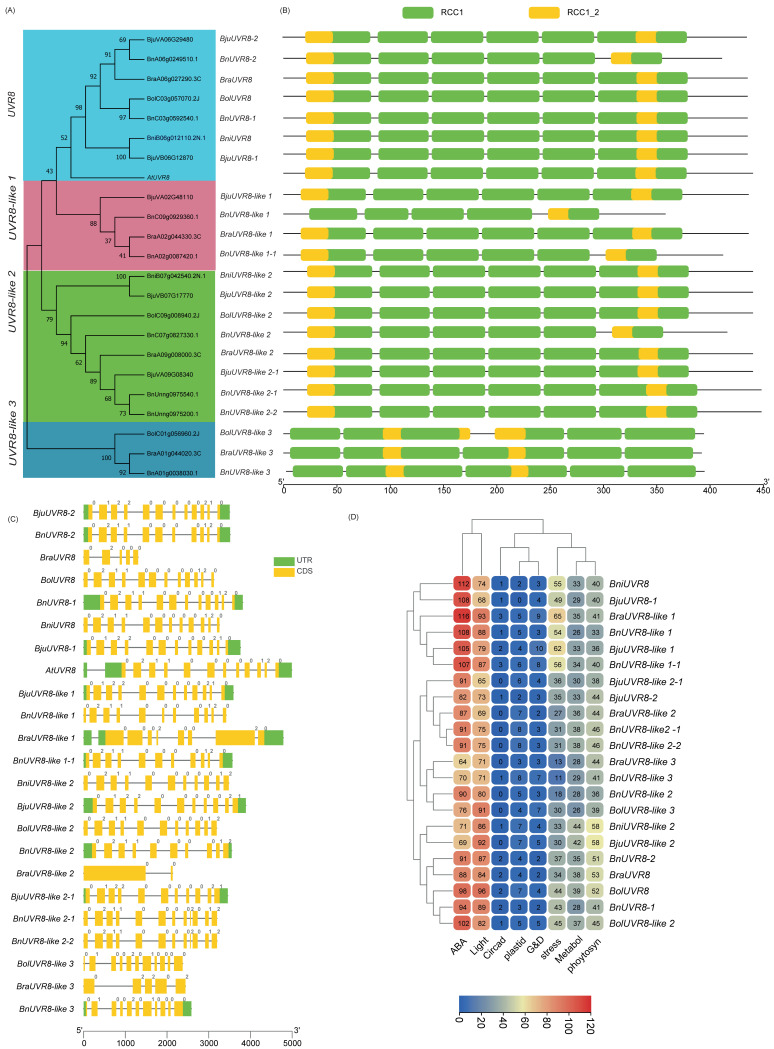
Phylogenetic analysis, classification, and structural description of UV-resistance locus 8 (UVR8) gene family in Brassica species. (**A**) The unrooted phylogenetic tree was established with Arabidopsis and five Brassica species (*B. rapa*, *B. oleracea*, *B. nigra*, *B. napus*, and *B. juncea*) UVR8 protein sequences using the neighbor-joining tree method with 1500 bootstrap replications in MEGA11. (**B**) Represents the domain architecture of phylogenetically classified UVR8 genes of Brassica species, while (**C**) represents the gene structures including Intron/exon pattern and intron phases (Class 0, 1, and 2) and UTRs. (**D**) Heatmap represents the key cis-regulatory elements (ABA- and light-responsive, elements associated with plastids, circadian rhythm, growth and development (G&D), stress, metabolite biosynthesis, and photosynthesis) and their abundance in putative promoter motifs of Brassica UVR8 genes reported in this study.

**Figure 5:**
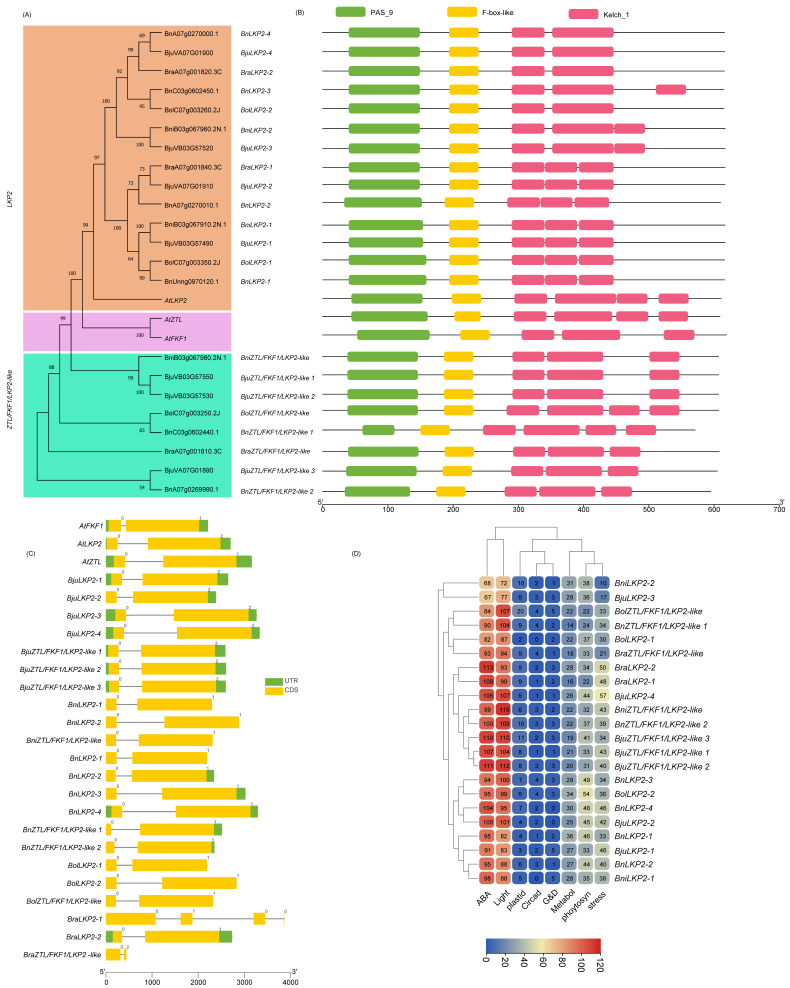
Phylogenetic analysis, classification, and structural description of F-box containing flavin binding protein (ZTL/FKF1/LKP2) gene family in Brassica species. (**A**) The unrooted phylogenetic tree was established with Arabidopsis and five Brassica species (*B. rapa*, *B. oleracea*, *B. nigra*, *B. napus*, and *B. juncea*) ZTL/FKF1/LKP2 class protein sequences using the neighbor-joining tree method with 1500 bootstrap replications in MEGA11. (**B**) Represents the domain architecture of phylogenetically classified ZTL/FKF1/LKP2 genes of Brassica species, while (**C**) represents the gene structures including Intron/exon pattern and intron phases (Class 0, 1, and 2) and UTRs. (**D**) Heatmap represents the key cis-regulatory elements (ABA- and light-responsive, elements associated with plastids, circadian rhythm, growth and development (G&D), stress, metabolite biosynthesis, and photosynthesis) and their abundance in putative promoter motifs of Brassica CRY genes reported in this study.

To identify the PHRs in five Brassica species, we performed BLASTP followed by conserved domain (CD) analyses against the local Brassica protein database (E-value < 1 × 10^−5^) by using the PHR protein sequences of *A. thaliana* as queries (Table S1). Most BLASTP hits to PHY, CRY2, CRY1, CRY3, ZTL/FKF1/LKP2, UVR8, PHOT1, and PHOT2 members had 62–94.7% AA sequence similarities. Few other BLASTP hits later identified as BnPHOT1-1, BolCRY3, BolUVR8-like 3, and BjuCRY3-1 with AA sequence similarity ranging from 30–37.6% had the requisite CD, were included in this study. Two significant AtCRY2 BLASTP hits with 89–92% AA similarity (BjuVB03G00710 and BolC09g006670.2J) had only one of two CDs (e.g., N-terminal photolyase domain was absent) were excluded for subsequent phylogenetic analysis/classification. Also, three AtUVR8 BLASTP hits identified as BolC02g061830.2J(C2), BolC02g061820.2J, and BniB05g025420.2N.1 were not considered for further study due to the absence of characteristic RCC1 and/or RCC1_2 domains. After the redundant sequences were removed, a total of 37, 37, 26, 22, and 22 putative members of CRY, PHY, PHOT, UVR8, and ZTL/FKF1/LKP2, respectively, were used for phylogenetic classification and further analysis. An unrooted phylogenetic tree was constructed for CRY (Figure 1), PHY (Figure 2), PHOT (Figure 3), UVR8 (Figure 4), and ZTL/FKF1/LKP2 (Figure 5) in concert with their Arabidopsis homologues.

**Figure 6:**
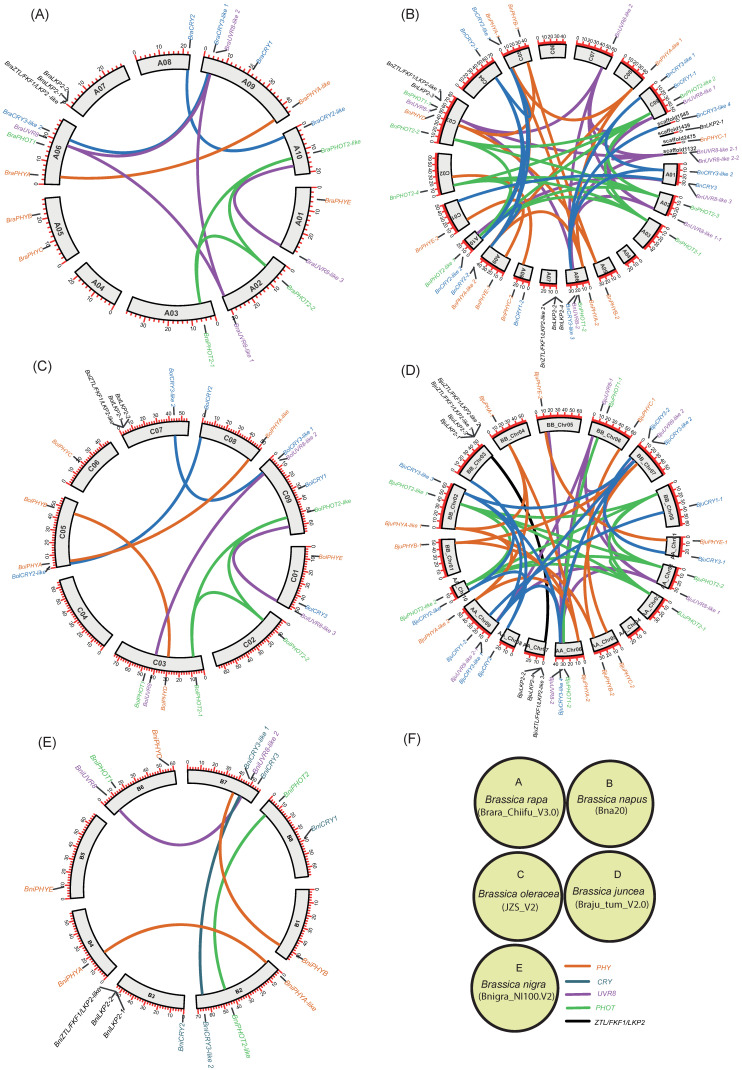
The chromosomal distribution and duplication of photoreceptor genes in Brassica species. (**A**–**E**) The Circos represent the chromosome maps (in Mbs) of *B. rapa* (**A**), *B. napus* (**B**), *B. oleracea* (**C**), *B. juncea* (**D**), and *B. nigra* (**E**). (**F**) describes the reference genomes of Brassica species (**A**–**E**) utilized for syntenic analyses while the different color schemes indicate the photoreceptor gene families.

The phylogenetic tree analyses revealed that the CRY, PHY, UVR8, and ZTL/FKF1/LKP2 proteins were clustered into 5, 6, 4, and 2 subfamilies/groups, respectively. Similarly, PHOT proteins had two main subfamilies with an additional seven individual outliers, which were designated as a PHOT2-like subfamily. All of the PHR proteins that had been clustered were named based on their phylogenetic relationship consistent with the Arabidopsis PHR proteins. Also, the phylogenetic outliers (i.e., Protein sequences that are not clustered with any of the AtPHR subfamilies) were renamed according to their phylogenetic relationships with other identifiable members and designated as a new subfamily. All of the Brassica CRY, PHY, PHOT, UVR8, and ZTL/FKF1/LKP2 gene families had one or more new subfamilies. The chromosomal distribution of the Brassica PHR genes and their syntenic relationships were presented in Circos plots (Figure 6; Table S2). The chromosomal mapping of Brassica PHR showed that they are located in all chromosomes with the exception of A04 (of *B. rapa*)*,* A04 and C06 (of *B. napus*), C04 (of *B. oleracea*), and AA_Chr04 (of *B. Juncea*). Also, the chromosomal distribution pattern of PHR genes of the diploid parents (*B. rapa* and *B. nigra*) were conserved in the A-, B- subgenome of *B. juncea* except for BB_chr08 and AA_chr01. This also reveals that segmental and tandem duplicates of PHR genes were ancient and originally from diploid parents. However, the chromosomal distribution pattern of PHR genes in *B. napus* and its diploid parents was not similar for most of the genes, possibly indicating the occurrence of evolutionary events posthybridization. Apart from that, there were PHR members found in four scaffolds of the *B. napus* genome.

### 2.2. History of Replication Retention and Gene Loss Events in Photoreceptor Genes in Brassica Species

We performed gene loss and replication retention analysis to elucidate the evolution of the photoreceptor genes in Brassica. We obtained quantitative changes in the number of genes in subfamilies based on the phylogenetic reconstruction (Table 1). For diploid Brassica, one *A. thaliana* gene should theoretically correspond to three genes, but there are different amounts of gene loss (fractionation) ranging from zero to three. The whole-genome triplication (WGT) event in the Brassica genus contains three diploids *B. rapa* (AA, n = 10), *B. oleracea* (CC, n = 9), *B. nigra* (BB, n = 8), and allopolyploids like *B. napus* (AACC, n = 19) and *B. juncea* (AABB, n = 18) theoretically should result in more gene copies than that of *A. thaliana* orthologues. However, the systematic identification and classification of Brassica PHR genes identified 0–2 gene copies in diploids and 0–4 in tetraploids. For instance, CRY1, 2, and 3 subfamilies had one copy in diploids and 2 in their tetraploids. However, the new CRY3-like subfamily had a maximum of 2 and 4 in di- and tetraploid species. Interestingly, the CRY3 subfamily was lost in *B. rapa*, or it might have evolved as a CRY3-like subfamily. In case of the PHY gene family, PHYD is not present in both A and B genomes of di- and tetraploid species, while all other subfamily members, including new PHYA-like, were observed (Table 1).

In total, 5–6 PHYs in diploids and 10–11 PHYs in tetraploids were identified. Among ZTL/FKF1/LKP2 members, only LKP2 and ZTL/FKF1/LKP2-like members were observed in Brassica species. Another family, PHOT, comprised of three subfamilies, namely PHOT1, 2, and PHOT2-like, which were present in all species. All the parental diploids have one member, PHOT1, like AtPHOT1. Unlike PHOT1, PHOT2 has retained duplicates in *B. rapa* and *B. oleracea*, albeit *B. nigra* remained with a single copy. The UVR8 phylogenetic classification added three new subfamilies (UVR8-like 1, 2, and 3) to this gene family. Of these, the members of UVR8 like-1 in *B. oleracea,* UVR8-like 3 in *B. nigra* and in *B. juncea* were not found in this study. The key observation is that the diploid *B. nigra* and tetraploid *B. juncea* unusually share the same number of gene copies for UVR8. *B. napus* had the largest UVR8 family consisted of eight members.

Whole-genome duplication (WGD), segmental/tandem duplication, inversions, and translocations are driving forces of genome evolution that increase genome plasticity. Based on the gene copy number and their distribution pattern in a single genome, they can be classified as singletons, dispersed duplicates, proximal duplicates, tandem duplicates, and segmental/WGD duplicates [24]. According to [24], we determined the evolutionary events/gene types in Brassica PHR gene families. In this study, 9 gene pairs each from *B*. *rapa* and *B. oleracea*, 4 pairs of *B. nigra*, 46 pairs of *B. napus*, and 12 pairs of *B. juncea* were the results of segmental duplication, thus altogether accounting for 94.73% of the total duplication events in PHR gene families (Table S3; Figure 6). On the other hand, one pair of tandem duplication each from *B. rapa*, *B. oleracea*, and *B*. *juncea*, and two tandem duplications from *B. napus* were observed. It is worth noting that tandem duplication was observed only in ZTL/FKF1/LKP2 gene families in Brassica, with the exception of *B. nigra*, where no tandem duplicates was observed. The tandem duplicates share 78 to 92 AA similarities and are adjacent paralogues on the same/single chromosomes. The calculated Ka/Ks ratio for the duplicates ranging from 0.03 to 0.40 shows that the aforementioned genes were under strong purifying selection. The lowest Ka/Ks ratio (0.03) was observed for BjuPHOT2-like 2: BjuPHOT2-like 1, while another gene pair (BnUVR8 like 2: BnUVR8 like 2-1) had the maximum Ka/Ks ratio of 0.40. The tandem duplication in BnLKP2-4: BnLKP2-2 and BjuLKP2-4: BjuLKP2-2 might have occurred 10-10.76 MYA while the other tandem pairs such as BnZTL/FKF1/LKP2-like 1: BnLKP2-3, BolZTL/FKF1/LKP2-like: BolLKP2-2, and BraZTL/FKF1/LKP2 -like: BraLKP2-2 seems to be ancestral tandem duplicates as inferred from their estimated divergence times of 14. 94–15.55 MYA. There were two members, BnUVR8-like 2-2: BnUVR8-like 2-1 (BnUnng0975200.1: BnUnng0975540.1) origins from scaffold1132, classified as segmentally duplicated genes had 100% AA similarity between them; hence the Ka/Ks ratio and divergence times could not be calculated. The BnCRY2-like 2: BnCRY2-like 1 is the youngest member (3.47 MYA) of the Brassica CRY family, followed by BnCRY2-2: BnCRY2-1 (4.4 MYA), BjuCRY1-2: BjuCRY1-1 (5.6 MYA), and BnCRY3-like 2: BnCRY3-like 1(6.2 MYA). Similarly, BnPHYB-2: BnPHYB-1 (3.95 MYA)*,* BjuPHYC-2: BjuPHYC-1 (4.8 MYA)*,* BnPHYE-1: BnPHYE-2 (4.9 MYA), BjuPHOT1-2: BjuPHOT1-1 (3.9 MYA)*,* BnPHOT2-3: BnPHOT2-4 (4.1 MYA)*,* BnPHOT2-like 1: BnPHOT2-like 2 (4.2 MYA), BnUVR8-like 2: BnUVR8-like 2-2 (3.1 MYA)*,* BnUVR8-like 2: BnUVR8-like 2-1 (3.1 MYA)*,* BnUVR8-2: BnUVR8-1(3.7 MYA)*,* BnUVR8-like 1-1: BnUVR8-like 1 (4.7 MYA) and BjuLKP2-4: BjuLKP2-2 (10 MYA) are the youngest member of their respective families. Whereas BnCRY2-like 2: BnCRY2-1 (16 MYA), BraPHYA: BraPHYA-like (19.9 MYA), BraPHOT2-2: BraPHOT2-1 (10.8 MYA), BnUVR8-like 2: BnUVR8-like 1 (12.7 MYA), and BjuZTL/FKF1/LKP2-like 3: BjuLKP2-3 (16.8 MYA) might be the oldest segmental duplicates of their respective families. As reported for the expansin superfamily [25], segmental duplication occurs most frequently in PHR gene families of Brassica species.

As demonstrated by [25], we also investigated the effect of hybridization events by comparing the number of PHR genes lost between parental diploids and their hybrids/tetraploids in this study. Gene losses or divergence in CRY, PHY, and PHOT of *B. napus* and *B. juncea* were directly reflected in parental diploids. For example, two copies were lost in the CRY1 subfamily of *B. rapa* and *B. oleracea*, which accounted for 4 lost copies in tetraploid *B. napus*. This trend was also true for PHY and PHOT members. However, the expansion or gene losses in UVR8 and ZTL/FKF1/LPK2-like subfamily of *B. napus* and *B. juncea* are not originally from their diploid parents and possibly the effects of hybridization events.

### 2.3. Domain Architecture and Physiochemical Properties of PHRs

The Brassica LKP2 protein might have lost one or two Kelch_1 domain at the third and fourth positions at C-terminal ends during evolution, while PAS_9 and F-box-like domains were present as in Arabidopsis LKP2 homologues (Table S4, Figure 5B). BnLKP2-4, BjuLKP2-4, and BraLKP2-2 lost two Kelch_1 (third and fourth), while BnLKP2-3 lost the third Kelch_1 domain. BniLKP2-2, and BjuLKP2-3 lost 4th Kelch_1 domain. The second positioned larger Kelch_1 domain in BraLKP2-1, BjuLKP2-2, BnLKP2-2, BniLKP2-1, BjuLKP2-1, BolLKP2-1, and BnLKP2-1 was replaced with a small third positioned Kelch_1 domain. BolZTL/FKF1/LKP2-like is the only member of Brassica that has similar domain composition to that of AtZTL and AtFKF1 proteins. Although BnZTL/FKF1/LKP2-like has similar domain compositions, the length of PAS_9 was shortened. The AtUVR8 has 4 individual RCC1 domains and a RCC1 and RCC1_2 coupled domain at both N- and C-terminal ends. The Brassica UVR8 domain compositions are structurally similar to AtUVR8 except BnUVR8-2, in which one of the coupled domains was incomplete at the C-terminal end. Apart from that, 15 Brassica proteins had similar domain compositions to UVR8; however, the number of domains or lengths or positions are slightly different, hence designated as Brassica UVR8-like proteins for the first time in this study. Of these, one coupled domain was completely lost in BnUVR8-like1 otherwise most of the UVR8-like 1 and UVR8-like 2 members were near similar to UVR8. However, UVR8-like 3 members do not have RCC1_2 at their original positions. Also, the second and third positioned RCC1 domains were integrated into a single/hybrid domain. Brassica CRY1,2,3 domains were similar to Arabidopsis, while the newly identified CRY2-like and most of CRY3-like were similar to CRY2 and 3 except BnCRY3-like3, BolCRY3-like 2, and BnCRY3-like 4 has an additional AdoMet_MTase domain at C-terminal ends. Similarly, most Brassica PHOT members have similar domain compositions, positions like AtPHOT members except for some members of *B. napus* (BnPHOT1-1, BnPHOT2-2, BnPHOT2-2, and BnPHOT2-like 2) do not have one of two PAS_9 domains present in other PHOT members. In the PHY family, PHYB members have an additional domain composed of HATPase except for BniPHYB. PHYD, PHYE, PHYC, and PHYA have similar domain architecture to Arabidopsis homologues. In the new subfamily PHYA-like, BnPHYA-like 2 have lost HATPase domain at C-terminal end while all other members structurally similar to PHYA-like or PHYA subfamily.

CRY2-like (~575AA) were shorter in AA length than CRY2 subfamily members (~623AA); hence the molecular weight and the hydrophilic coefficient of CRY2-like were smaller than CRY2 while the pI ranges between 5.56 to 5.99. Both CRY2 and CRY2-like were localized to the chloroplast (Table S5). Unlike AtCRY3, which was localized to chloroplast and mitochondria, Brassica CRY3 members were predicted to localize only in the chloroplast, while all the CRY3-like members were likely to present in chloroplast and mitochondria. In general the Brassica CRY family has 40 members distributed across 5 species, BnCRY3-like 4 being the largest in terms of length (813 AA), while the shortest one was also from the same subfamily, i.e., BnCRY3-like 1 (516 AA). All Brassica PHY members, including newly reported PHYA-like members, were likely destined to work in the nucleus with ~1131 AA and pI ranging from 5.55 to 6.29. similarly, ZTL/FKF1/LKP2 members localized to the nucleus while the pI of ZTL/FKF1/LKP2-like members was higher than others. The PHOT members are likely to be present in cell membranes at cellular levels. Of these three members, BraPHOT1, BjuPHOT1-1, and BjuPHOT2-1 were likely to be present in the nucleus in addition to cell membranes. The mean AA length is ~911 AA (lowest-727, highest-996), and the pI varies between 6.19 to 8.7. Unlike other PHR genes, the UVR8 members were found with single to multiple cellular localization signals, most of which have nucleus localization signals followed by cell wall and cytoplasm. Distinctly, BolUVR8-like 3 was predicted to present in the Golgi apparatus in addition to the cytoplasm and nucleus.

### 2.4. Gene Structure and Promoter Motifs Analysis

**Figure 7:**
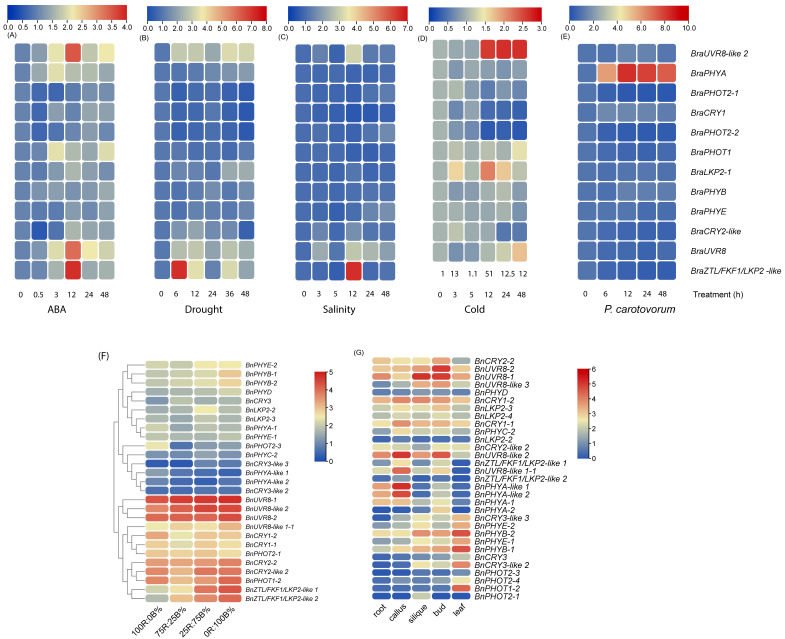
Transcriptional expression changes of photoreceptor (*PHR*) genes in stress conditions and in different tissue. (**A**–**E**) Heatmap representing the microarray-derived expression pattern (Log2FC) of *B. rapa* PHR transcripts in response to exogenous ABA, abiotic stress conditions (drought, salinity, and cold), and *Pectobacterium* *carotovorum* infections. (**F**,**G**) The RNA-seq data derived expression changes [Log2(FKPM)] of *B. napus* treated with different ratios of red (R)- and blue (B)-LED light conditions and in different tissue of *B. napus* (root, callus, silique, bud, and leaf).

The structural comparison of two or more gene of a single family will aid in understanding the evolutionarily structural changes/gene diversification that occurred in those genes. The distribution patterns of the 45 of 143 Brassica PHR exon/introns (e.g., number of exons, order and their types (symmetrical and asymmetrical)) were similar to that of Arabidopsis homologues, indicating these genes were evolutionarily conserved. This includes three UVR8 genes (*BolUVR8*, *BnUVR8-1*, and *BniUVR8*), all genes of the ZTL/FKF1/LKP2 family except *BraLKP2-1*, nine members of PHY (*BniPHYA*, *BnPHYA-1*, *BolPHYA*, *BnPHYB-1*, *BolPHYD*, *BnPHYD*, *BniPHYA*, *BnPHYA-1*, and *BolPHYA*), four members of PHOT (*BolPHOT1*, *BniPHOT1*, *BolPHOT2-1*, and *BniPHOT2*), and eight of CRY (*BnCRY2-2*, *BolCRY2*, *BnCRY2-1*, *BniCRY2*, *BolCRY2-like*, *BnCRY2-like 1*, *BnCRY2-like 2*, and *BnCRY1-1*). At the levels of gene family, the intron/exon organization of *PHY*, *ZTL/FKF1/LKP2* were fairly conserved over the members of other PHR gene families. In terms of species, *B. napus* genes were largely conserved over other species. It is worth noting that the PHR genes of *B. rapa* were the least conserved among others. This could be majorly contributed to *BraLKP2-1*, *BraUVR8*, *BraUVR8-like 1*, *BraUVR8-like 2*, and *BraUVR8-like 3* having 4, 5, 8, 2, and 5 exons, respectively, against 2,12,12,12,12 of their orthologues. Similarly, the structure of *BraPHYA-like* looks different from others with 10 exons, including 3 asymmetrical exons. *BraPHYC* has 6 instead of 3 exons. Brassica *PHOTs* have 19–22 exons except for *BraPHOT1*, *BraPHOT2-2*, and *BraPHOT2-like* had 4, 1, and 4 exons, respectively. *BraCRY3-like 1* has just an exon against 12 of *AtCRY3* and Brassica genes belonging to this family, while another *BraCRY1* had 6 against 4. Among CRY subfamilies, *CRY2* was largely conserved over others. Based on the presence of the number of introns, the PHR genes can be classified into two types: a gene that contains no or one intron, and the gene that contains multiple introns. *BraCRY3-like 1* has no introns, while *BraUVR8-like 2*, *BraPHOT2-2*, and all of the ZTL/FKF1/LKP2, with the exception of *BraLKP2-1*, contain just one intron. Most other genes contain 2–22 introns, and the PHOT gene family has the highest number of introns among other PHR gene families. *PHOTs* had symmetrical exon (0,0 class) at the 3′ end with exception of *BraPHOT2-2* and *BraPHOT2-like*. Conversely, except for *BraLKP2-1*, all of the ZTL/FKF1/LKP2 genes have asymmetrical exons (0,1 or 0,2) at the 3′ end. The tandem duplicated gene pairs (Figure 7) have structural similarities between them and are conserved for exon/intron organization and intron phases, with the exception of *B. rapa* genes.

The promoter motifs/cis-regulatory elements of a gene can determine their regulatory pathways/downstream gene expression in response to perceived internal and external signal cues in plants. Therefore, we used in silico promoter analysis for Brassica PHR genes. The results showed they are enriched with several motifs, including ABA, light, plastid, circadian, growth and development, metabolites, photosynthesis, and stress-related. By abundance, the ZTL/FKF1/LKP2 gene family has the largest number (~96.63 motifs) of light response motifs, followed by PHY, PHOT, CRY, ZTL, and UVR8. Interestingly the ranking of ZTL/FKF1/LKP2 was also true to ABA response motifs. Metabolites, stress, growth, and development-related motifs were relatively higher in PHOT members than in other PHR genes. Similar analysis revealed that PHY has the highest number of motifs associated with plastids and photosynthesis. In comparison with UVR8-like genes, UVR8 genes had a markedly larger mean number of ABA and stress-related motifs. Similarly, the promoters of UVR8, and PHY had higher average for ABA and stress-related motifs than UVR8-like and PHY-like subfamilies. In contrast, CRY-like subfamilies average number of ABA and stress motifs were higher than their classical subfamilies. However, PHOT and PHOT-like subfamilies have almost similar motif compositions.

### 2.5. Expression Profiling of PHR Genes in Different Tissue and Stress Conditions

Due to several ABA-, stress-associated promoter motifs in *PHR* genes, we investigated the microarray-based expression profiles of 12 *B. rapa PHR* genes in the presence of ABA hormones or stress conditions like drought, salinity, cold, and *P. carotovorum* infections at different time intervals. True to the enriched promoter motifs, most of the genes were differentially expressed under ABA and stress conditions. *BraUVR8-like 2*, *BraPHYA*, *BraPHOT1*, *BraLKP2*, *BraUVR8*, and *BraZTL/FKF1/LKP2-like* genes were upregulated by those treatments at one or more intervals. *BraZTL/FKF1/LKP2-like* was upregulated during abiotic stress conditions, especially under cold stress highest upregulation was observed. *BraPHYA* was the only gene that showed consistent upregulation under *P. carotovorum* infection while most other genes were downregulated. Under salinity, *BraPHYA*, *BraPHOT2-1*, *BraCRY1*, *BraPHOT1*, and *BraPHYB* were consistently downregulated. By contrast, none of the genes was consistently downregulated under ABA presence.

The RNA seq data-based B. napus tissue-specific expression analysis revealed that BnPHYB-2, BnPHYB-1, BnCRY3-like 2, and BnPHOT1-2 of leaves, BnUVR8-1, BnUVR8-2, and BnUVR8-like2 of buds, BnUVR8-1 of silique, BnPHYA-like1,2, BNUVR8-like1-1, and BnUVR-like2 of callus were highly expressed (Figure 7). In contrast, the levels of expression of BnCRY3, BnCRY3-like2, BnPHOT2-1,3,4, BnPHYA-1,2 and BnLKP2-2 of roots, BnPHO2-1, BnPHOT2-3, BnPHYA-2 of callus, BnPHYA-like 2, BnZTL/FKF1/LKP2-like 2, and BnLKP2 of silique, BnLKP2-2, BnPHOT2-1 of buds, BnZTL/FKF1/LKP2-like-1,2, BnUVR8-like 1-1, BnPHYA2, BnPHOT2-1 of leaves were relatively lower than others. Of these, the expression of BnPHOT2-1 was lower with the exception of siliques. As expectedly, blue LED or compound light (25R:75B) induced the expression of BnZTL/FKF1/LKP2-like 1,2, BnPHOT1-2, BnCRY2-like 2, and BnCRY2-2. However, blue light did not induce the expression of some other blue light photoreceptor genes like BnCRY3-like 3 and BnCRY3-like 2. Similarly, the expression of some red light photoreceptor genes BnPHYA-like 1,2, and BnPHYC-2 were not greatly influenced by red light. Interestingly, UVR8 family genes (BnUVR8-1,2, BnUVR8-like2, BnUVR8-like 1-1), BnCRY2-2, BnCRY2-like 2, and BnPHOT1-2 were induced at all light conditions (100R:0B, 75R:25B, 25R:75B and 0R:100B%). The expression of BnCRY3-like 3, BnCRY3-like 2, and BnPHYA-like 2 was lower in similar light conditions.

## 3. Discussion

Plants utilize PHRs to monitor almost all facets of light (e.g., intensity, direction, duration, and wavelength of light) to optimize their growth and development. Plant PHRs consisted of specific photoreceptive domains that are useful in perceiving, interpreting, and transducing light signals to nuclei, where transcriptional reprogramming takes place, which ultimately offers developmental plasticity in plants [26,27,28]. Before this study, there was no systematic study done for the identification of PHR genes in Brassica species. Therefore, in the present study, we used systematic approaches at genomic scales to identify and understand the post-WGT molecular evolutionary events of CRY, PHY, PHOT, UVR8, and ZTL/FKF1/LKP2 gene families in five important Brassica species. A total of 21, 22, 21, 44, and 39 PHR genes comprised of five gene families were identified in *B. rapa*, *B. oleracea*, *B. nigra*, *B. napus*, and *B. juncea*, respectively. The phylogenetic reconstruction of PHR members of Brassica added several new subfamilies, including CRY2-like, CRY3-like, PHYA-like, PHOT2-like, UVR8-like 1,2,3, and ZTL/FKF1/LKP2-like. Of these, CRY3-like and UVR8-like 2 constitute the largest subfamily in their respective gene families. The new subfamily members mostly had phylogenetic relationships and similar domain compositions to that of classical subfamilies, albeit with different domain positions and lengths. In some cases, they do have additional or lost CDs. e.g., PHYB subfamily members had an additional domain belonging to the HATPase superfamily, with the exception of BniPHYB. Whereas BnPHYA-like 2 had lost an HATPase domain, it was still included in the PHYA-like subfamily, as it meets other requisite domains and does not have an additional domain; hence it is not likely to consider as a non-photoreceptor family protein.

Genomic replication often results in the expansion of the gene family [20,21,25]. Therefore, to understand the quantitative changes that occurred in the PHR gene family following WGT in Brassica, we obtained the quantitative changes in the number of gene copies for each subfamily. Following WGT, every diploid Brassica species theoretically should contain three Arabidopsis homologues and six copies in tetraploid Brassica species. However, none of the Brassica PHR members contains the aforementioned copies in any of the tetraploids or their progenitors, suggesting PHR genes were lost on a large scale, possibly due to functional redundancy between the replicates after the triploidization. The large-scale loss/genome contraction was also noted for other gene families like *AHLs*, *EXPs,* and flowering-time genes in Brassica [25,29,30]. In particular, heavy gene losses have occurred for CRY3 and PHYD subfamilies in all the Brassica species. In PHYD, there were no copies found in *B. rapa* (A genome), *B. nigra* (B genome) and *B. juncea* (A and B genome). In contrast, a single copy was noted in both *B. oleracea* (C genome) and *B. napus* (A and C genome), suggesting PHYD is possibly required only in species with the C genome. An interesting observation is that PHOT1, CRY1,2, PHYA, PHYB, PHYC, and PHYE subfamilies do not retain replicates in diploid genomes following WGT and maintain single-member subfamily just like *A. thaliana*. In ZTL/FKF1/LKP2, only LKP2 retained duplicates while the other two members lost completely. Moreover, all diploids had an additional, single-member PHYA-like subfamily; the function of this subfamily is yet to be known. The expression profiling of *BnPHYA-like 1* and *BnPHYA-like 2* are not greatly influenced under red- and compound- LED light conditions. Interestingly, both genes were more abundantly present in callus, root, and buds than in leaf and silique tissue (Figure 7), possibly suggesting a key role in the growth and development of callus, root, and buds in *B. napus*.

As demonstrated by [25], we also investigated the effect of hybridization events by comparing the number of PHR genes lost between parental diploids and their hybrids/tetraploids in this study. The evolutionary histories (e.g., gene loss/replication retention) of CRY, PHY, and PHOT gene families in parental diploids were highly conserved in their hybrids like *B. napus* and *B. juncea*. For example, both *B. rapa* and *B. oleracea* lost two copies during evolution, attributable to 4 CRY1 copies lost in tetraploid *B. napus*. This trend indicates that there was no gene loss/retention following hybridization. However, the same was not true for UVRB or ZTL/FKF1/LKP2 gene families, thus revealing posthybridization evolutionary changes in tetraploids occurred for UVR8 and ZTL/FKF1. In fact, the single-member *AtUVR8* homologues greatly expanded/diverged (three new subfamilies) more than any other PHR gene family in Brassica. More specifically, the BniUVR8 gene family has relatively more gene copies than other diploids. Surprisingly, only LKP2 of ZTL/FKF1/LKP2 was noted in Brassica. The functional redundancy between the family members might result in the loss of ZTL and FKF1 members, as reviewed by [3].

Also, differences in exon/intron organizations can clarify the molecular evolution of the gene family [31]. Of the 19 subfamilies reported here, only seven (PHYD, PHYC, UVR8, CRY2, CRY2-like, LKP2, ZTL/FKF1/LKP2 like) have a similar number of exon/introns with the exception of some *B. rapa* members suggesting those subfamilies were highly conserved. However, most other subfamilies have a different number of exons/introns suggesting a high proportion of *PHR* genes were structurally diversified during evolution. Studies show that the intron phase determines exon shuffling potential and ultimately protein domain shuffling during protein evolution [32] which could offer novel functionalities. In this study, the distribution of intron phases is nonuniform within or between PHR subfamilies with a high proportion of phase 0 introns (highest mean) except PHY. Despite shared histories of common ancestors, this phase distribution pattern in Brassica species suggests extensive structural diversification through shuffled exons [33], possibly attributable to lineage specificity. The *B. rapa* PHR gene members were structurally different from other Brassica species. Of these, BraCRY3-like 1 has no introns while BraUVR8-like 2, BraPHOT2-2, and all of the ZTL/FKF1/LKP2 with the exception of BraLKP2-1, contain just one intron, which is different from other subfamily members. In general, PHOT members had symmetrical exons (0,0 class) at the 3′ end, while ZTL/FKF1/LKP2 members have asymmetrical exons (0, 1 or 0, 2) at the 3′ end. The significance of the presence of a symmetrical exon is that it can be shuffled by intronic recombination without disrupting the reading frame [34]. Except in *B. rapa*, the exon/intron numbers and intron phases were conserved in tandem duplicates.

To understand the biological significance or to infer information on potential transcriptional regulators, we analyzed promoter motifs for their association with some biological processes. PHR promoter motifs are generally enriched with several motifs, including ABA, light, plastid, circadian, growth and development, metabolites, photosynthesis, and stress-related, possibly indicating PHRs respond to multiple signals, including abiotic stresses, thus affirming their role as “multisensory proteins.” Interestingly, ZTL/FKF1/LKP2-like genes had a markedly larger mean number of ABA, and light-related motifs to ZTL/FKF1/LKP2 subfamily members, indicating some uniqueness to ZTL/FKF1/LKP2-like genes firstly reported in this study.

Like EXP [25], AHL [29], and Homeobox (HB) gene families of Brassica, segmental duplication is the driving force in the expansion of the PHR gene families. The most recent segmental duplication occurred in *B. napus* (for BnUVR8 like 2: BnUVR8 like 2-1, BnUVR8 like 2: BnUVR8 like 2-2) was 3.7 MYA. Regardless of the species, segmentally duplicated gene pairs of PHY undergo a strong purifying selection than that of segmental/tandem duplicated gene pairs found in ZTL/FKF1/LKP2, CRY, UVR8, and PHOT members. This strong purifying selection would be the reason that PHYA, PHYB, PHYC, and PHYE subfamilies do not retain replicates and maintain single-member subfamilies in diploid genomes. Moreover, in tetraploids, the expression pattern between duplicated gene pairs such as BnPHYB-1: BnPHYB-2, BnPHYE-1: BnPHYE-2, and BnPHYA-like 1: and BnPHYA-like 2 in response to differential light qualities and in different tissue are similar (Figure 7) indicating the possible functional redundancy between them, which also can be another reason for their strong negative selection. Although there were several tandem duplicates found in Brassica species [35], only ZTL/FKF1/LKP2 was found with tandem duplicates among all the PHR gene families in Brassica species in this study.

In conclusion, this is the first comprehensive and systematic study to identify 144 photoreceptor genes in Brassica species. This comprehensive study reported several new subfamilies, including CRY2-like, CRY3-like, PHYA-like, PHOT2-like, UVR8-like 1, 2, 3, and ZTL/FKF1/LKP2-like based on their phylogeny with Arabidopsis homologues. The molecular evolution of PHR showed indirect expansion and gene losses at a large scale. In particular, PHYD was completely lost in the A and B genomes and retained only in the C subgenome. In comparison, the UVR8 was markedly expanded than other PHR gene families in Brassica. Segmental duplication at large and tandem at small scales are responsible for expanding PHR gene families. All of the PHR genes undergo purifying selection, yet relatively stronger selection was observed in PHY genes. The presence of several important cis-elements in PHR promoter motifs and the expression of some PHR genes in the presence of exogenous ABA, differential light, stress conditions, and tissue-specific expression patterns suggest that PHRs are important in multiple biological processes. Collectively, this study provides basic resources for functional characterization of PHR genes, which will be helpful in Brassica crop-improvement programs to harness the desired agronomic traits.

## 4. Materials and Methods

### 4.1. Identification of Photoreceptor Gene Families from Brassica Species

The genomic resources of *Brassica rapa*, *Brassica napus*, *Brassica oleracea, Brassica juncea,* and *Brassica nigra* are available at BRAD (http://brassicadb.agridata.cn/brad/ (accessed on 29 April 2022)) were used in this study. The reference protein sequences of five Brassica species were concatenated (copy *.fas combinedfile.fas) to create a single protein database (makeblastdb -in db.fasta -dbtype prot -out db) in NCBI standalone BLAST tool to facilitate Local BLASTP with Arabidopsis query sequences. The Arabidopsis CRY (AtCRY1_ NP_567341.1; AtCRY2_ NP_849588.1; AtCRY3_ NP_568461.3), PHY (AtPHYA_ NP_172428.1; AtPHYB_ NP_001325249.1; AtPHYC_ NP_198433.1; AtPHYD_ NP_193360.1; AtPHYE_ NP_193547.4), PHOT (AtPHOT1_ NP_190164.1; AtPHOT2_ NP_001318824.1), UVR8 (AtUVR8_ NP_201191.1) and AtZTL/FKF1/LKP2 (AtZTL_ NP_568855.1; AtFKF1_ NP_564919.1; AtLKP2_ NP_849983.1) members were used as query for the identification of Brassica CRY, PHY, PHOT, UVR8, and ZTL/FKF1/LKP2 gene family members, respectively. BLASTP was carried out with “blastp -query query.fa -db db -outout.txt -outfmt “6 qseqid qlen sseqid salltitles pident mismatch gapopen qstart qend qcovs sstart send evalue bitscore” -evalue 0.00001 -max_target_seqs 20-num_threads 4” command. The BLAST hits were evaluated for the characteristic conserved domains of the respective gene family by the CD-search tool [36]. The search was set against Pfam v33.1-18271 PSSMs with 0.010000 as the expected value threshold. The significant domain hits were visualized by Tbtools v1.09873 (Toolbox for Biologists) [37] using the “Visualize NCBI CDD Domain pattern” tool. The presence of characteristic CD and its orders were considered for further selection. The BLAST hits with the required CD in the right order as that of queries with or without additional domains were selected even if the sequence similarity between them is less than 80%, and the rest were discarded for further analyses. The “ProtParam” tool [38] was used to obtain the physical and chemical properties of Brassica PHR proteins. Additionally, the subcellular localization of PHR proteins was predicted using Plant-mPLoc webtool [39,40,41,42,43].

### 4.2. Phylogenetic Analysis and Classification of Photoreceptor Gene Family/Subfamily Members

The multiple BLASTP hits with CDs of each gene family and Arabidopsis queries were aligned using the MUSCLE alignment tool of MEGA11 [44]. The alignment options were kept as default, with UPGMA as the cluster method for each gene family. The unrooted phylogenetic tree was constructed using the neighbor-joining tree method with 1500 bootstrap replications, and Poisson as substitution model. The other parameters were default. The classification was based on the phylogenetic clustering of Brassica sequences with Arabidopsis subfamily members/homologues. A subfamily’s single-copy gene gets the name of Arabidopsis homolog present in the same clusters with the exception of the initials of its species of origin. Additionally, a serial number was placed after the gene name as per the phylogenetic ranking if multiple gene copies are present in a subfamily. The phylogenetic outliers were designated as novel/new subfamily, and the gene nomenclature is partly obtained from possibly closest phylogenetic clusters.

### 4.3. Gene Duplication, Synteny, Selection Pressure Analysis

Tbtools v1.09873 was used to investigate gene duplication events and the syntenic relationship between members of a particular Brassica photoreceptor gene family. Briefly, the protein sequences of *B. rapa*, *B. napus*, *B. oleracea*, *B. juncea*, and *B. nigra* downloaded from the BRAD database were individually self-BLASTED by the “Blast several Sequences to a Big Database.” The parameters set were Outfmt: Table; NumofThreads: 2; E-value: 1 × 10^−5^; NumofHits: 5 and NumofAligns: 5. Meanwhile, the Gff gene annotation file for each genome was preprocessed/simplified with “File Merge For MCScanX” with Merge mode set to “GtfGff2SimGxf.” MCScanX was executed by “Quik Run MCScanX Wrapper” [24] for each genome separately to find collinearity genes and gene types (tandem/segmental/proximal/dispersed) at the genomic scale. The resultant gene collinearity information fed to “File Merge For MCScanX” (merge mode: collinear) to identify gene pairs/duplicated genes of desired gene family. The linked regions from gene pairs and respective merged Gff files were utilized to identify synteny through “File Transformat for MicroSynteny Viewer.” The chromosome features of all five species were derived from individual gff annotation files, and the syntenic relationships of selected genes were visualized through “Advanced Circos” of Tbtools. The “simple Ka/Ks Calculator (NG)” was utilized to infer the synonymous (Ks) and nonsynonymous (Ka) nucleotide substitutions in tandem or segmentally duplicated genes and their ratios (Ka/Ks) to measure the selection pressures (Ka/Ks > 1 (positive) or Ka/Ks < 1 (purifying)) during evolution. According to [45], the divergence time (MYA, million years ago) of duplicated genes was calculated with the formula T = Ks/2r, where r is 1.5 × 10^−8^ synonymous substitutions per site per year, and it is the rate of divergence for nuclear genes from plants.

### 4.4. Promoter Motif Analysis

To get the putative promoter regions of each PHR gene family of Brassica, the genome sequences and gff annotation files of respective Brassica species downloaded from the BRAD database were used. To extract 2 kb upstream bases of each gene reported in this study, “Gtf/Gff3 Sequence extract” under GTF/GFF3 manipulate command of Tbtools v1.09873 was utilized. GFF3 and genome of Brassica species were separately fed as input; parameters were set to extract 2 kb bases upstream of the transcription start site of each gene and requested to retain only upstream bases. The resultant putative promoter sequences of selected genes were separated, and random manual verification was done to confirm the promoter sequences of desired genes. For cis-acting element analysis, the online tool New PLACE (https://www.dna.affrc.go.jp/PLACE/?action=newplace) (accessed on 9 May 2022) [46] was used. The abundance of putative promoter motifs belonging to key biological processes was represented by heatmap using Tbtools.

### 4.5. Gene Structure Analysis

The gene structure of each PHR family was visualized by the “Gene Structure View (Advanced)” of Tbtools [37]. The tree layout was set to the origin, and the Intron phase number was included to know the distribution of symmetrical and asymmetrical exons for understanding the evolutionarily structural changes.

### 4.6. Expression Profiling of Photoreceptor Genes

The plant material, growth conditions, treatments, and cDNA synthesis for microarray experiments were originally from a previous study [47]. Briefly, the leaves of *B. rapa* cv. Chiifu inbreed seedlings (3-week-old) grown in soil-containing plastic pots, maintained at standard growth conditions (16 h/8 h photoperiods, 25 °C temperature, and 70% relative humidity), were sprayed with abscisic acid (ABA) (100 μM)/NaCl (250 mM; salt)/polyethylene glycol 6000 (4% *w*/*v*; drought)/*Pectobacterium carotovorum* suspension (6.15 log_10_ colony-forming units/mL) and whole plants were sampled at different intervals. Similarly, for inducing cold stress, plants were kept at 4 °C and sampled. Plants grown at standard growth conditions without chemical/stress agents/mocked samples (e.g., 0.2% Tween 20 and 0.1 DMSO for ABA) were designated as control and collected simultaneously with respective treated samples. Samples in triplicates for each treatment were used for total RNA extraction, cDNA synthesis, and subsequent analysis with microarray. The microarray hybridization reaction and data analysis were performed by GGBIO (http://www.ggbio.com) according to the manufacturer’s instructions (NimbleGen Inc., Madison, WI, USA; GenePix scanner 4000B (Axon, Scottsdale, AZ, USA)). The relative expression changes (Log2FC) of PHR transcripts in comparison with controls (0 h) were calculated and presented as heatmap. Additionally, the normalized expression values of *PHRs* (FPKM values) in *Brassica napus* var. *napus* (cv. zhongshuang 11) treated with differential LED light qualities (red (100%), blue (100%), and compound lights (75R:25B or 75B:25R)) were extracted from the Brassica Expression DataBase (BrassicaEDB), v1.0 [48] and log2[FPKM] values of *BnPHRs* were used to generate a heatmap with Tbtools. Also, the expression changes of *BnPHRs* across five different tissue types (root, calli, silique, bud, and leaf) were extracted from the public database (https://bnaomics.ocri-genomics.net/tools/exp-view/tissue.php) (accessed on 4 June 2022) and presented as a heatmap.

## Figures and Tables

**Table 1:** Expansion and contraction of photoreceptor gene families in Brassica species.

Gene Family	Subfamily	*Arabidopsis*	*B. rapa*	*B. oleracea*	*B. nigra*	*B. napus*	*B. juncea*
*CRY*	*CRY1*	1	1 (L2)	1 (L2)	1 (L2)	2 (L4)	2 (L4)
	*CRY2*	1	1 (L2)	1 (L2)	1 (L2)	2 (L4)	1 (L5)
	*CRY3*	1	0 (L3)	1 (L2)	1 (L2)	1 (L5)	2 (L4)
	*CRY2-like*	-	1	1	0	2	1
	*CRY3-like*	-	2	2	2	4	4
Total		3	5	6	5	11	10
*PHY*	*PHYA*	1	1 (L2)	1 (L2)	1 (L2)	2 (L4)	2 (L4)
	*PHYB*	1	1 (L2)	1 (L2)	1 (L2)	2 (L4)	2 (L4)
	*PHYC*	1	1 (L2)	1 (L2)	1 (L2)	2 (L4)	2 (L4)
	*PHYD*	1	0 (L3)	1 (L2)	0 (L3)	1 (L5)	0 (L6)
	*PHYE*	1	1 (L2)	1 (L2)	1 (L2)	2 (L4)	2 (L4)
	*PHYA-like*	-	1	1	1	2	2
Total		5	5	6	5	11	10
*PHOT*	*PHOT1*	1	1 (L2)	1 (L2)	1 (L2)	2 (L4)	2 (L4)
	*PHOT2*	1	2 (L1)	2 (L1)	1 (L2)	4 (L2)	3 (L3)
	*PHOT2-like*	-	1	1	1	2	2
Total		2	4	4	3	8	7
*UVR8*	*UVR8*	1	1 (L2)	1 (L2)	2 (L1)	2 (L4)	2 (L4)
	*UVR8-like 1*	-	1	0	1	2	1
	*UVR8-like 2*	-	1	1	2	3	2
	*UVR8-like 3*	-	1	1	0	1	0
Total		1	4	3	5	8	5
*ZTL/FKF1/LKP2*	*ZTL/FKF1/LKP2*	1/1/1	0/0/2 (L3/L3/L1)	0/0/2 (L3/L3/L1)	0/0/2 (L3/L3/L1)	0/0/4 (L6/L6/L2)	0/0/4 (L6/L6/L2)
	*ZTL/FKF1/LKP2-like*	-	1	1	1	2	3
Total		3	3	3	3	6	7

Numbers represent the number of Arabidopsis homologues observed in each species at the subfamily level. The “L” indicates gene loss and the number after “L” represents the number of gene copies lost after the triploidization event.

## Data Availability

All the necessary datasets are provided in the main text and in the supplementary data sections.

## Supplementary Materials

The following supporting information can be downloaded at: https://www.mdpi.com/article/10.3390/ijms23158695/s1.
